# Transcriptome profiling of spike provides expression features of genes related to terpene biosynthesis in lavender

**DOI:** 10.1038/s41598-020-63950-4

**Published:** 2020-04-24

**Authors:** Danli Guo, Kaicheng Kang, Pu Wang, Min Li, Xianzhong Huang

**Affiliations:** 1 Institute of Agricultural Sciences, Fourth Division of Xinjiang Production and Construction Corps, Cocodala, 835213 People’s Republic of China; 2 Special Plant Genomics Laboratory, College of Life Sciences, Shihezi University, Shihezi, 832003 People’s Republic of China; 3 College of Agriculture, Anhui Science and Technology University, Fengyang, 233100 People’s Republic of China

**Keywords:** Plant sciences, Secondary metabolism

## Abstract

Lavender (*Lavandula angustifolia*) is an important economic plant because of the value of its essential oil (EO). The Yili Valley in Xinjiang has become the largest lavender planting base in China. However, there is a lack of research on the gene expression regulation of EO biosynthesis and metabolism in local varieties. Here, *de novo* transcriptome analysis of inflorescence of three development stages from initial flower bud to flowering stage 50% from two lavender cultivars with contrasting EO production revealed the dynamics of 100,177 differentially expressed transcripts (DETs) in various stages of spike development within and across the cultivars. The lavender transcriptome contained 77 DETs with annotations related to terpenoid biosynthesis. The expression profiles of the 27 genes involved in the methylerythritol phosphate (MEP) pathway, 22 genes in the mevalonate (MVA) pathway, 28 genes related to monoterpene and sesquiterpene biosynthesis during inflorescence development were comprehensively characterized, and possible links between the expression changes of genes and contents of EO constituents were explored. The upregulated genes were mainly concentrated in the MEP pathway, while most genes in the MVA pathway were downregulated during flower development, and cultivars with a higher EO content presented higher expression of genes in the MEP pathway, indicating that EOs were chiefly produced through the MEP pathway. Additionally, MYB transcription factors constituted the largest number of transcripts in all samples, suggesting their potential roles in regulating EO biosynthesis. The sequences and transcriptional patterns of the transcripts will be helpful for understanding the molecular basis of lavender terpene biosynthesis.

## Introduction

The *Lavandula* genus (*Lamiaceae*) includes over 39 species, and three of these species are mainly cultivated to extract essential oil (EO): *Lavandula latifolia* (spike lavender), *Lavandula angustifolia* (fine lavender, or lavender), and their hybrid *Lavandula* × *intermedia* (lavandin)^1,2^. EOs have great economic value, as they are widely used in cosmetics and health care products^3,4^. Plant breeding has broadened the variety of types; and varieties, climate conditions and extraction methods not only determine the essential composition of the EO itself, but also the market value of EO^5^. Lavender EO is a mixture of monoterpenes (C_10_) and sesquiterpenes (C_15_), which occurs mainly in the glandular trichome secretory cells covering the surfaces of leaves and floral tissues^6,7^. Of the various monoterpenes in EO, linalool and linalyl acetate are the two major constituents^5^.

In plants, EO components are synthesized by the condensation reaction of the universal isoprenoid precursor isopentenyl pyrophosphate (IPP) and its double-bond isomer dimethyl allyl pyrophosphate (DMAPP)^8,9^ through the mevalonate (MVA) and the 2-C-methyl-D-erythritol 4-phosphate (MEP) pathways, also called the 1-deoxy-D-xylulose 5-phosphate (DXP) pathway^10–12^. In the MEP pathway, the first intermediate is DXP, which is synthesized from glyceraldehyde-3-P (G3P) and pyruvate, through a reaction catalysed by 1-deoxy-D-xylulose-5-phosphate synthase (DXS) that is considered to be a transcriptional controlled regulatory step^13^. The enzymes needed for the next six steps, DXP reductoisomerase (DXR), 2-C-methyl-D-erythritol 4-phosphate cytidylyltransferase (CMS), 4-(cytidine 5′-diphospho)-2-C-methyl-D-erythritol kinase (CMK), 2-C-methyl-D-erythritol 2,4-cyclodiphosphate synthase (MCS), (E)-4-hydroxy-3-methyl-but-2-enyl pyrophosphate synthase (HDS), and (E)-4-hydroxy-3-methyl-but-2-enyl pyrophosphate reductase (HDR) in plastids, are involved in the formation of IPP/DMAPP.

IPP/DMAPP are also synthesized by six step-wise enzymes via the cytosolic MVA pathway, starting from the thiolysis of acetyl-CoA (AAC) by AAC thiolase (AACT). The MVA pathway intermediate products and enzymes are 3-hydroxy-3-methylglutaryl-CoA (HMG-CoA), which is catalysed by HMG synthase (HMGS); MVA, which is catalysed by HMG reductase (HMGR), a key enzyme of this pathway^13^; mevalonate-5-phosphate (MVAP), which is catalysed by the MVA kinase (MK); mevalonate-5-pyrophosphate (MVAPP), which is catalysed by the phosphomevalonate kinase (PMK); and IPP, which is catalysed by the mevalonate 5-pyrophosphate decarboxylase (MVD)^14,15^. In a particular plant cell part, IPP and DMAPP are converted to a C_10_ compound geranyl pyrophosphate (GPP) and a C_15_ compound farnesyl pyrophosphate (FPP) by prenyl pyrophosphate synthases^16^. Subsequently, mono- and sesquiterpenes are synthesized by GPP and FPP, respectively, via a series of terpene synthases (TPSs).

Considerable research has been undertaken to understand the biosynthetic pathways of EOs. Numerous genes that encode TPSs and contribute to EO production have been described in different lavender species, including *DXS*, *DXR*, (*S*)*-linalool synthase* (*sLiS*), (*R*)*-linalool synthase* (*rLiR*) and *alcohol acetyltransferase* (*AAT*)^2,17–23^. To sum up, the efforts of scientists have made substantial progress in understanding the synthetic and metabolic mechanisms of EOs in lavender. However, due to the influence of variety and environmental cues on EO synthesis, it is necessary to further explore the mechanism of EO biosynthesis from different perspectives using plant species growing under different phenological conditions. RNA-Seq is an economical method for deep sequencing of plant transcripts, as well as an effective way to study gene regulation including the identification of genes with low expression^2,24,25^. Furthermore, in the SRA database, there were fewer raw sequencing reads for lavender (*L*. *angustifolia*) RNA-Seq (https://www.ncbi.nlm.nih.gov/sra/?term=Lavandula). Lavender inflorescence has been suggested to be a model to study regulation of terpenes synthesis^26^. The characterization of gene expression changes during different development stages by RNA-Seq can be used to explore the synthesis and metabolism of terpenoids.

Each plant EO contains more than 100 compounds, and the content of each component is not fixed. The composition of lavender EO varies greatly due to the variations of plant varieties, growth period, water volume, altitude, geographical distribution, temperature, etc. Xinjiang is located in Northwest China and occupies one-sixth of the total territory of China. Xinjiang is divided into northern and southern regions by the Tianshan Mountains, and the climate in the North is quite different from that in the South. In the northern region, the amount of yearly rainfall is comparatively greater than that in the southern region. The Yili Valley is located north of Xinjiang, at a northern latitude of 42°–44°and 600–800 metres above sea level, surrounded by mountains on three sides. The climate and soil conditions in the Yili Valley are very similar to those of the world-famous lavender origins in Provence, France and Hokkaido, Japan. Lavender was introduced into Yili from France in 1964, and has become one of the major economic crops in the region. By the end of 2014, the area of lavender planting in Yili reached approximately 3333.33 ha. The Yili Valley is the largest producer of lavender in China and one of the four largest lavender gardens in the world, therefore it is called the “Oriental Provence” and “the hometown of lavender in China”^1^.

To our knowledge, there is no comparative transcriptome analysis of cultivars at different step of floral development in Xinjiang lavender. In this study, *de novo* sequencing of the transcriptome at three spike developmental stages, followed by annotation, expression profiling and validation were carried out in two *L. angustifolia* cultivars. The RNA-Seq data will provide a resource for comparative genomics and can help identify novel genes related to EO biosynthesis and metabolism in lavender plants.

## Results

### The difference in the contents of EO between *L. angustifolia* cultivar Xinxun 2 and YXA-5

**Figure 1:**
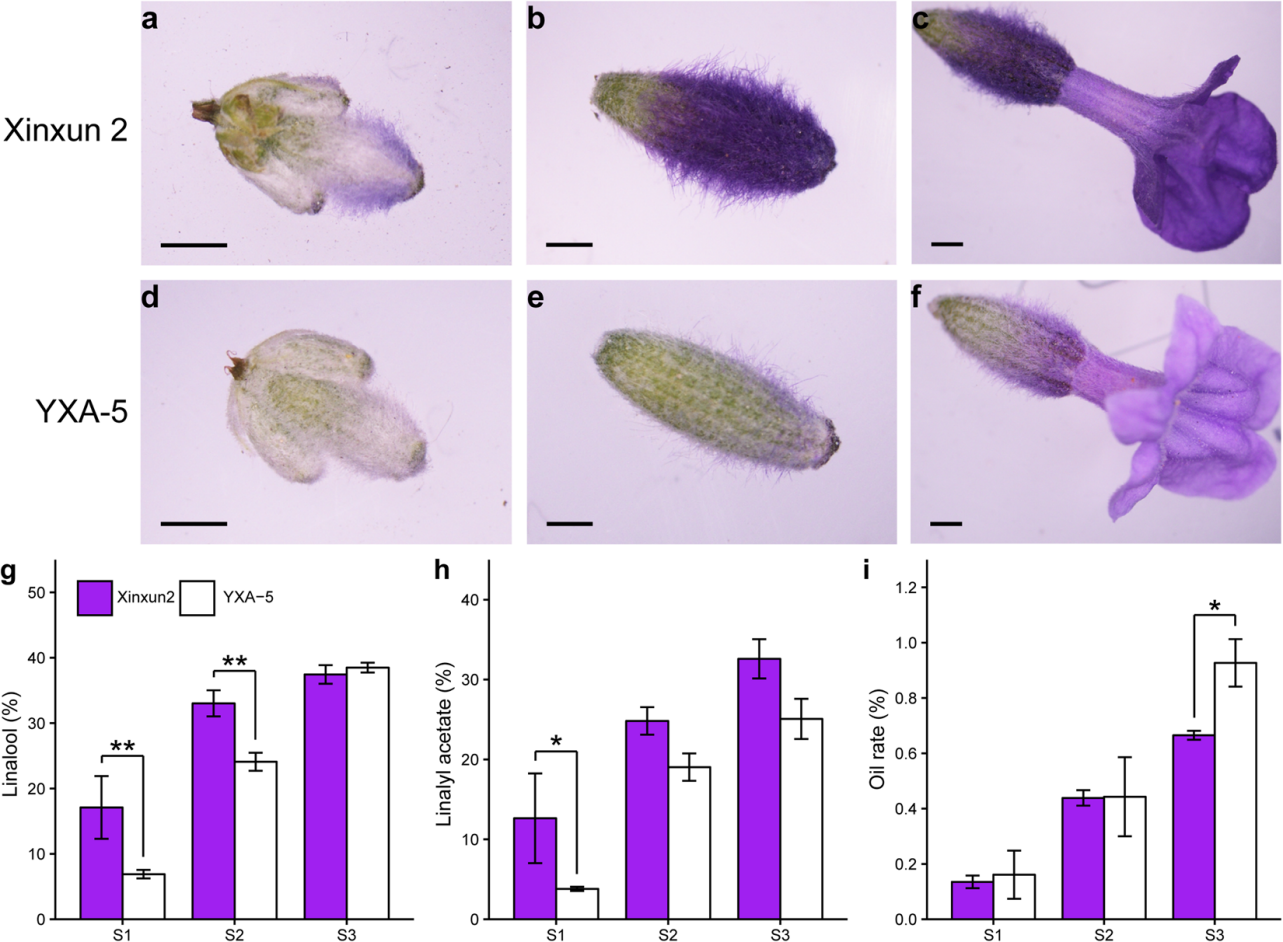
Flowering stages and variations in the EO content in different developmental stages of spikes in two lavender cultivars. Flowering stage Bud I (**a,d**). Flowering stage Bud II (**b,e**). Flowering stage 50%, where 50% of the spikes are in bloom (**c,f**). Bar = 1 mm. Levels of linalool (**g**), linalyl acetate (**h**), and oil rate (**i**) at the three developmental stages in the spikes of *L. angustifolia*. S1–S3 represent Bud I, Bud II, and Flowering stage 50% stages, respectively. Data represent the mean ± SE obtained from three independently biological replicates. Asterisks in (**g–i)** indicate significant differences (Student’s *t*-test, *P < 0.05 and **P < 0.01).

To more precisely determine the differences in EOs between the two *L. angustifolia* cultivars, a gas chromatography-mass spectrometry (GC-MS) approach was used. We analysed the production of EOs in two varieties at three developmental stages (Fig. 1a–f) and mainly measured the linalool and linalyl acetate content and oil rate (Fig. 1g–i). The content of each terpenoid was calculated as percentage of the total EOs. Oil rate was calculated by dividing the oil weight by the fresh weight. As shown in Fig. 1, except for in the flowering stage 50%, the linalool content in the Bud I and Bud II stages of Xinxun 2 were higher than those of YXA-5 (Fig. 1g). In the Bud I stage, the linalyl acetate in Xinxun 2 was significantly higher than that of YXA-5 (Fig. 1h). The data showed that the white flowering lavender contained a higher oil rate than the purple flowering lavender at the flowering stage 50% (Fig. 1i). The colour of the spikes in the Xinxun 2 cultivar is purple, and therefore we designated the three development stages: PS1, PS2 and PS3. The colour of the spikes in YXA-5 is white, and therefore the three developmental stages were designated WS1, WS2 and WS3.

### RNA sequencing, *de novo* assembly, and annotation

**Figure 2:**
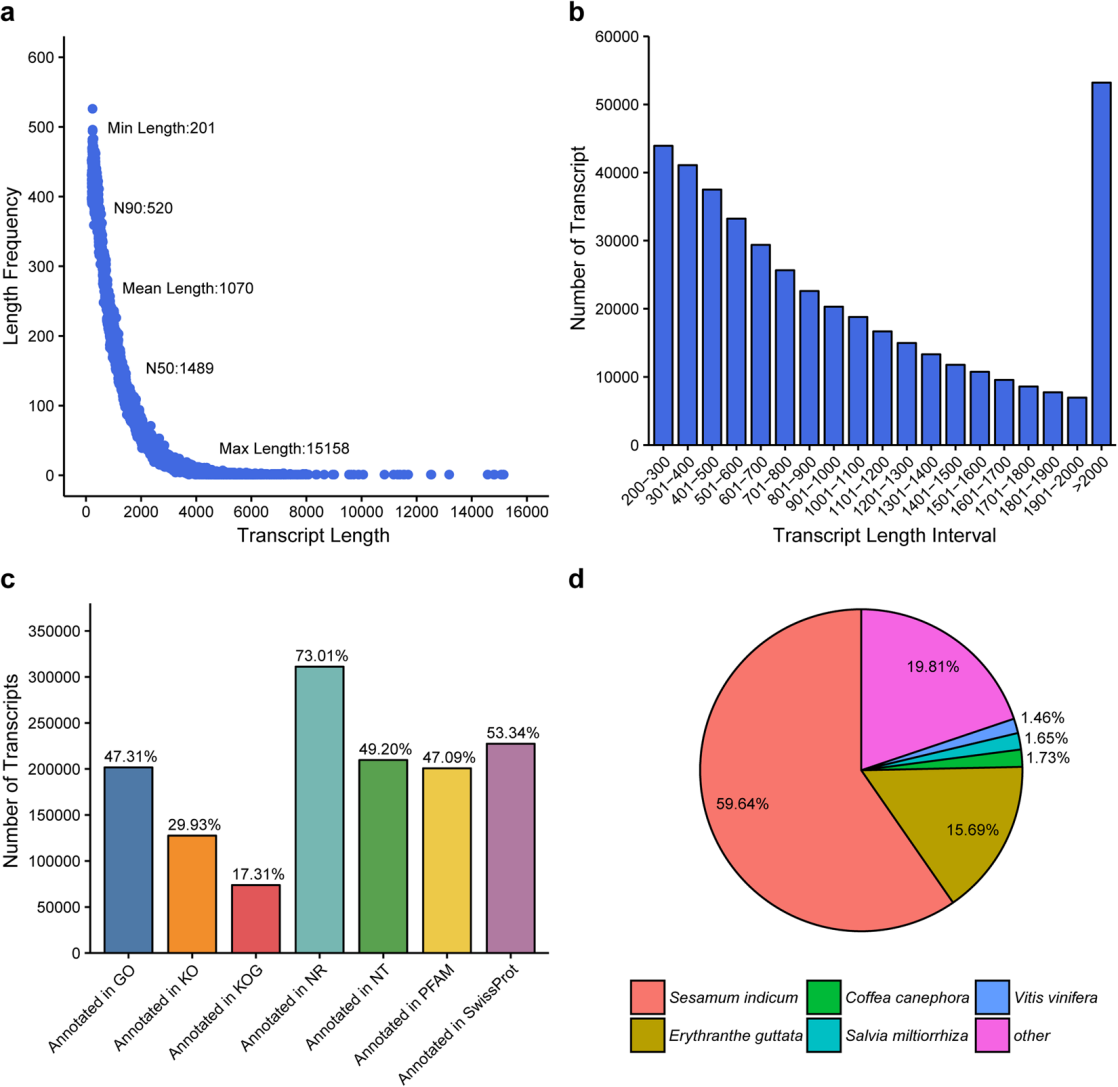
Illumina sequencing and transcriptomes of *L. angustifolia*. (**a**) Detailed information on the assembled unigenes. (**b**) Size distributions of the unigenes. (**c)** Annotation of the unigenes in seven various databases. (**d)** Species distribution of the annotated unigenes.

To explore the dynamics characteristics of gene transcription during inflorescence development, total RNA isolated from flower tissues of two *L. angustifolia* cultivars, Xinxun 2 and YXA-5, was sequenced via RNA-Seq technology to yield approximately 1030 million raw reads and 1015 million clean reads (Supplementary Table S1). *De* novo assembly of these clean reads using the Trinity program^25^ generated a large number of unique transcripts. To assess the completeness of the assembled *L. angustifolia* transcriptome, we performed BUSCO analyses by searching against the conserved 1440 plant-specific sequences (Embryophyta data set from BUSCO data sets)^27^. As shown in Supplementary Table S2, we found that 1064 (73.9%) transcripts had complete BUSCO hits, indicating the assembled transcriptome was well assembled with good quality. The size of the transcript varied from 201 bp to 15,158 bp (*N*_50_: 1489 bp), with a mean length of 1070 bp (Fig. 2a). The majority of the transcripts (59.5%) had 200 to 1000 bp lengths. The lengths of 27.9% transcripts ranged from 1000 and 2000 bp, and 12.6% transcripts were longer than 2000 bp (Fig. 2b). The number of transcripts annotated with seven public databases is summarised in Fig. 2c. A total of 311,224 (73.01%) and 227,392 (53.34%) transcripts received annotations from NCBI (https://www.ncbi.nlm.nih.gov/) non-redundant protein (NR) and Swiss-Prot databases (http://www.expasy.org/sprot/), respectively. Gene ontology (http://www.geneontology.org), protein family (http://pfam.xfam.org/) and Kyoto Encyclopedia of Genes and Genomes (http://www.genome.jp/kegg) annotation analysis indicated that 201,698 (47.31%), 200,757 (47.09%) and 127,605 (29.93%) transcripts matched these databases, respectively. The genomic similarity analysis by BLASTx showed that the most abundant transcripts were annotated as *Sesamum indicum*, which accounted for 59.6%, followed by *Erythranthe guttata* (15.7%); only 1.7%, 1.7% and 1.5% sequences had hit against in *Coffea canephora*, *Salvia miltiorrhiz* and *Vitis vinifera*, respectively; 19.8% of annotated sequences were similar to other plant species (Fig. 2d). A box diagram of fragments per kilobase of transcript sequence per million base pairs (FPKM) indicated that gene expression levels (FPKM > 0.3) were not evenly distributed at the three developmental stages (Supplementary Fig. S1). There was an obvious increase at the Bud I and Bud II stages compared to the flowering stage 50%.

### Identification and functional analysis of differentially expressed transcripts (DETs) during *L. angustifolia* flower development

**Figure 3:**
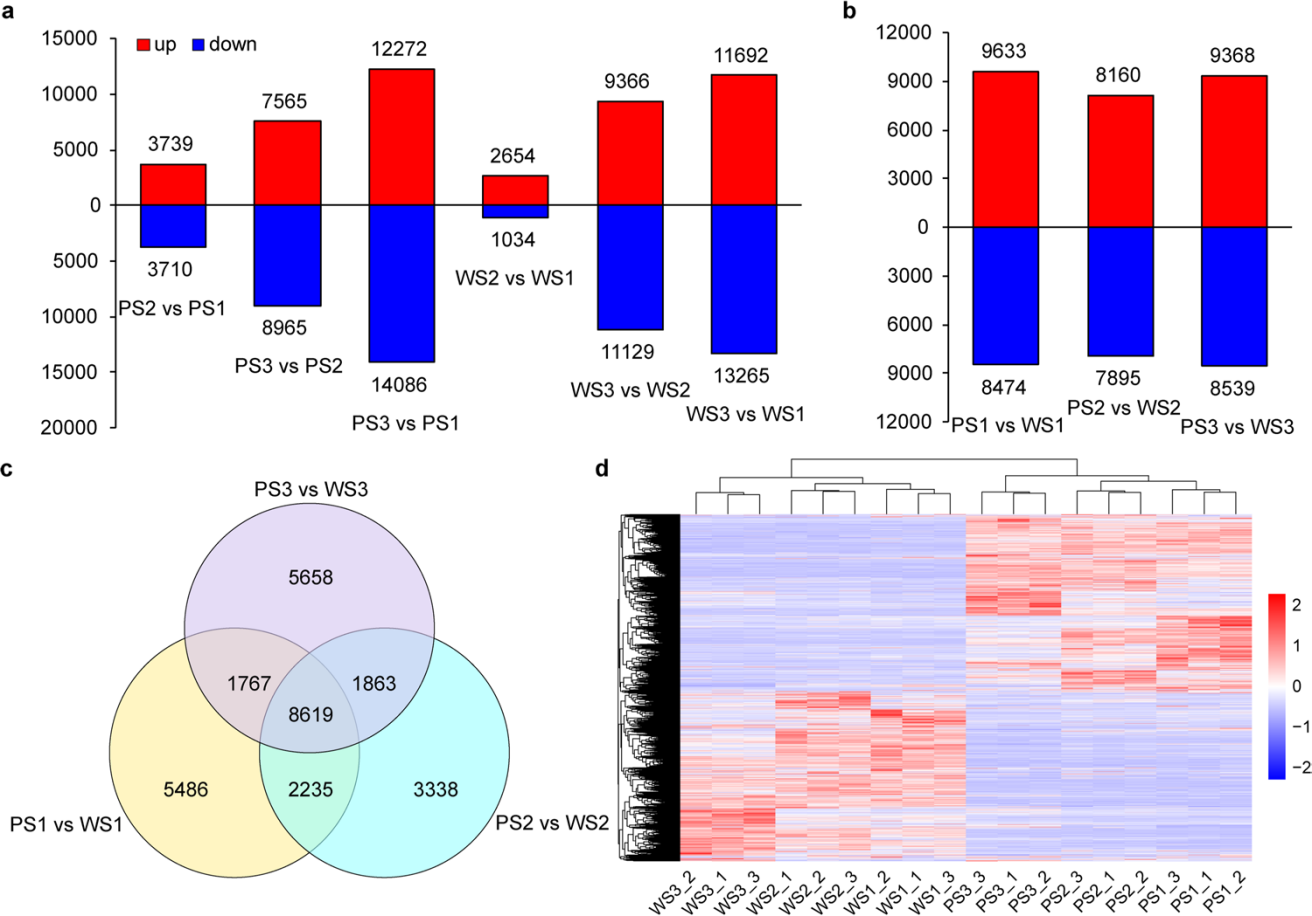
Characterization of differential gene expression in Xinxun 2 compared with YXA-5 at three developmental stages of the lavender spike. (**a**) The number of upregulated (upper bars) and downregulated (lower bars) genes at three stages of flowering in Xinxun 2 and YXA-5 cultivars, respectively. (**b)** Comparison of upregulated and downregulated genes at three stages of flowering between two *L. angustifolia* cultivars. (**c**) Venn diagram displaying the number of DEGs in each comparison between two cultivars. (**d**) Expression profiles and cluster analysis of DEGs at three developmental stages in *L. angustifolia* spikes in two cultivars. PS1, PS2, and PS3 indicate the purple lavender flowers at Bud I, Bud II, and flowering stage 50%, respectively. WS1, WS2, and WS3 indicate the white lavender flowers at Bud I, Bud II, and flowering stage 50%, respectively, and 1, 2, and 3 represent three independent biological replicates.

To investigate the differences in gene expression that characterise different developmental stages of lavender spikes, we first analysed the DETs between two developmental stages in two cultivars in the samples by using DESeq2 software (*P*-value < 0.05; Fig. 3a). The results show that Bud I stage in PS2/PS1 or WS2/WS1 contained the smallest number of DETs (7449 and 3688) in the purple or white flower cultivar comparison, respectively. The contents of linalool and linalyl acetate were lowest in Bud I, and were highest at flowering stage 50%, which was related to the amount of gene expression. The differences in gene expression at the flowering stage 50% were greatest. In Xinxun 2, 3739 and 12,272 genes were upregulated, and 3710 and 14,086 genes were downregulated in PS2 and PS3 compared with PS1, respectively. In PS3 vs PS2, 7565 genes were upregulated, and 8965 genes were downregulated. In YXA-5, 2654 and 11,692 genes were upregulated, and 1034 and 13,265 genes were downregulated in WS2 and WS3 compared with WS1, respectively. In WS3 vs WS2, the number of upregulated genes was 9366, and the downregulated genes were 11,129. These results indicated that the expression levels of genes coincided with the production of EOs (Fig. 1g–i). A comparison between two cultivars in the same period indicated that the number of upregulated DETs in each group was greater than the downregulated DETs (Fig. 3b). A total of 9633, 8160 and 9368 genes were upregulated in PS1 vs WS1, PS2 vs WS2 and PS1 vs WS1, respectively, whereas 8474, 7865 and 8539 genes were downregulated in these groups, respectively. Comparison of three developmental stages (PS1 vs WS1, PS2 vs WS2 and PS1 vs WS1), with more genes upregulated than downregulated, may lead to differences in terpenoid contents (Fig. 1g,h). Further analysis identified which of the DETs in each comparison were unique or shared across the three developmental stages. A Venn diagram (Fig. 3c) showed that a total of 8619 transcripts were shared across all three stages in two cultivars. The transcript profiles for the entire lavender spike DETs in two cultivars and between two cultivars were clustered to visualize these differences using hierarchical cluster analysis (Fig. 3d). These data of two lavender cultivars suggested that a dramatic change of gene expression would lead to significant changes in terpenoid contents, which was consistent with the determinant results of linalool and linalyl acetate in each cultivar during inflorescence development. Furthermore, the GO enrichment analysis of DETs revealed that “metabolic process” was the most categories of the biological process (Supplementary Fig. S2). KEGG pathway enrichment analyses of DETs in each comparison in the two cultivars unveiled that “terpenoid backbone biosynthesis” and “sesquiterpenoid and triterpenoid biosynthesis” were also highly enriched (Supplementary Table S3). These data indicated that the expression changes of genes associated with terpenoid metabolism were very active during inflorescence development in lavender.

### Differential regulation of genes associated with the EO synthesis pathway

**Figure 4:**
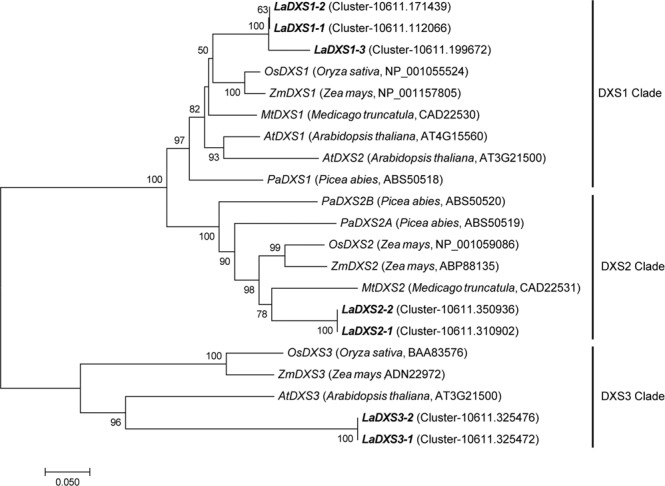
Phylogentic analysis of *DXSs* in *L. angustifolia*. A phylogenetic tree of plant *DXSs* based on the neighbor-joining method. Six *DXSs* from *L. angustifolia* were in bold.

**Figure 5:**
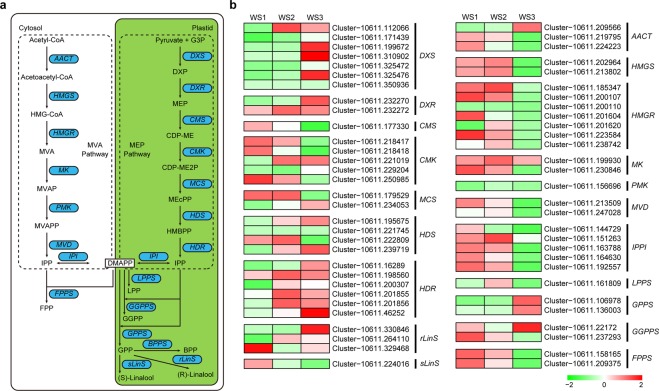
Differential expressions of the unigenes related to the essential oil (EO) biosynthesis pathway in YXA-5. (**a**) Schematic diagram of the terpene biosynthesis pathway. (**b)** Heatmap depicting the expression profile of genes related to EO biosynthesis in different reaction steps.

Because the production and quality of EO from lavender plants are the main concern, we carried out a comprehensive analysis of the genes related to the EO synthesis pathways. The putative genes associated with terpenoid biosynthesis were manually selected according to the KEGG annotation and tBLASTx search. A total of 77 genes with annotation relating to EO synthesis pathways were differentially expressed across three different stages of flower development examined in this study (Table 1). The detailed information on these differentially expressed genes (DEGs) was listed in Supplementary Table S4. In the MEP pathway, there is different *DXS* genes involved in the biosynthesis of terpene metabolites. The transcriptomics analysis revealed that a total of 7 unique putative genes related to *DXS* were differentially expressed during flower development. Phylogenetic analysis, using the lavender *DXS* genes identified in this study with other study, showed that lavender *DXS* family genes belonged the plant three class of *DXS* clade (Fig. 4). *LaDXS2-1* (cluster-10611.310902) and *LaDXS2-2* (cluster-10611.350936) belonged to *DXS2* clade, which was regarded as to be involved in the formation of isoprenoid metabolism^26^. In plants, monoterpenes are usually generated via the MEP pathway with seven enzymatic steps in plastids (Fig. 5a). Expression analysis revealed that most of *DXSs* were upregulated during inflorescence development in two cultivars, which exhibited a similar trend with terpenoid biosynthesis. Transcripts of *LaDXS2-1* and *LaDXS2-2* peaked at flowering stage 50% in respective cultivar, suggesting their important roles in EO biosynthesis. *LaDXS1-3* (cluster-10611.199672) and *LaDXS3-2* (cluster-10611.325476) expressions peaked at flowering stage 50% in YXA-5. Transcripts of *LaDXS1-3*, *LaDXS3-1* (cluster-10611.325472) and *LaDXS3-2* (cluster-10611.325476) were also significantly upregulated during inflorescence development in two cultivars (Fig. 5b and Supplementary Fig. S3). The transcriptome investigation identified two DEGs for *DXR*, one for *CMS*, five for *CMK*, and two for *MCS*. Four DEGs for *HDS* and six for *HDR* were also identified in this study. Expression analysis revealed that most of these genes were upregulated during inflorescence development. Interestingly, as a last gene for the MEP, all six *HDR* genes were significantly upregulated during flower development, reflecting more efficient in terpenoid biosynthesis.

**Table 1:** Summary of annotations of unigenes associated with essential oil-synthesis pathways in *L*. *angustifolia* transcriptome data.

Gene ID	Description	No. of DEGs
**Methylerythritol pathway (MEP)**		
*DXS*	1-deoxy-D-xylulose 5-phosphate synthase	7
*DXR*	1-deoxy-D-xylulose 5-phosphate reductoisomerase	2
*CMS*	2-C-methyl-D-erythritol 4-phosphate cytidylyltransferase	1
*CMK*	4-(cytidine 5′-diphospho)-2-C-methyl-D-erythritol kinase	5
*MCS*	2-C-methyl-D-erythritol 2,4-cyclodiphosphate synthase	2
*HDS*	(E)-4-hydroxy-3-methyl-but-2-enyl pyrophosphate synthase	4
*HDR*	(E)-4-hydroxy-3-methyl-but-2-enyl pyrophosphate reductase	6
**Mevalonate pathway (MVA)**		
*AACT*	Acetoacetyl-coa thiolase	3
*HMGS*	3-hydroxy-3-methylglutaryl-coa synthase	2
*HMGR*	3-hydroxy-3-methylglutaryl-coa reductase	7
*MK*	Mevalonate-5-kinase	2
*PMK*	Phosphomevalonate kinase	1
*MVD*	Mevalonate-5-pyrophosphate decarboxylase	2
*IPPI*	Isopentenyl pyrophosphate isomerase	5
**Terpene synthesis pathway**		
*LPPS*	lavandulyl diphosphate synthase	1
*GPPS*	geranyl diphosphate synthase	2
*GGPPS*	geranylgeranyl diphosphate synthase	2
*FPPS*	farnesyl diphosphate synthase	2
*rLinS*	(R)-linalool synthase	3
*sLinS*	(S)-linalool synthase	1
*PhilS*	*β*-phellanderene synthase	2
*CinS*	1,8-cineole synthase	4
*LimS*	limonene synthase	3
*GerS*	germacrene D synthase	4
*CadS*	cadinol synthase	2
*β-CaryS*	*β*-caryophyllene synthase	2

In plants, the MVA pathway provides precursors for cytosolic and mitochondrial isoprenoids including sesquiterpenes, sterols and ubiquinones^16^. In the MVA pathway, the initial product acetoacetyl-CoA is synthesized by two molecules of acetyl CoA catalysed by AACT, and HMGS catalyses the reaction of acetoacetyl-CoA with acetyl CoA to generate HMG-CoA (Fig. 5a). Three unigenes for *AACT* and two unigenes for *HMGS* were identified to be differentially expressed in the spike development of *L. angustifolia*. Furthermore, seven unique putative transcripts representing NADH-dependent enzyme HMGR in *L. angustifolia*, which catalyses the biosynthesis of MVA from HMG-CoA^13^, were observed to be differentially expressed. Phylogenetic analysis of *LaHMGR* and selected plant *HMGR* sequences revealed that four *LaHMGRs* had the closest relationship with *SiHMGR* (*Sesamum indicum*), and three *LaHMGR* had the closest relationship with *SsHMGR* (*Salvia splendens*) and *SmHMGR* (*Salvia miltiorrhiza*) (Supplementary Fig. S4). Expression analysis revealed that these seven *LaHMGRs* were universally downregulated at flowering 50%. MVA is transformed into MVAP through phosphorylation catalysed by MK, and then MVAP is converted into MVAPP catalysed by PMK. Thereafter, MVAPP is converted into IPP catalysed by MVD (Fig. 5a). Examination of the lavender transcriptome demonstrated that two *MK*, one *PMK* and two *MVD* putative genes were differentially expressed in the two cultivars in this study. Furthermore, these genes were also generally downregulated at flowering stage 50% (Fig. 5b; Supplementary Fig. S3).

IPP is converted into DMAPP through an isomerization reaction catalysed by isopentenyl pyrophosphate isomerase (IPPI), and IPP and DMAPP are two phosphorylated isoprenes for the biosynthesis of isoprene, such as mono, sesqui, di and triterpenes^28^. Five *IPPI* genes were identified in *L. angustifolia* transcriptome. One *IPPI* (cluster-10611.151236) was upregulated from Bud I to Bud II, and them significantly downregulated at flowering stage 50% in YXA-5. However, other four *IPPI* genes were downregulated at three developmental stages. In Xinxun-2, five *IPPI* were universally regulated at flowering stage 50% (Supplementary Fig. S3). Also, transcriptome profiling validated one representative unigenes for lavandulyl pyrophosphate synthesis (*LPPS*), two for geranylgeranyl pyrophosphate synthase (*GGPPS*), two for FPP synthase (*FPPS*), two DEGs for GPP synthase (*GPPS*) were differentially expressed during flower development in two *L. angustifolia* cultivars. Amino acid sequences alignment revealed that LPPS proteins have high similarity among *L. angustifolia* and selected plant LPPS sequences (Supplementary Fig. S5). Expression analysis revealed that transcript of *LPPS* peaked at Bud II, two *GPPS* were significantly upregulate at flowering stage 50%, and two *FPPS* were downregulated during flower development. Of the two *GGPPS*, one was upregulated, and anther is downregulated during flower development (Supplementary Fig. S3).

In *lavandula* EOs, linalool exists in two isomers, *R* and *S*^2^. Three transcripts for (*R*)-linalool synthase (*rLinS*), and one for (*S*)-linalool synthase (*sLinS*) were identified from two lavender cultivars. Expression analysis showed that of three *rLinS*, two were upregulated and their expression patterns paralleled linalool accumulation during inflorescence development; and one was significantly downregulated in YXA 5, but was weakly downregulated in Xinxun 2. Also, one (*S*)-linalool synthase (*sLinS*) was identified to be downregulated from Bud I to flowering stage 50% in two cultivars.

### Identification and expression profiles of putative transcription factor (TF) families

**Figure 6:**
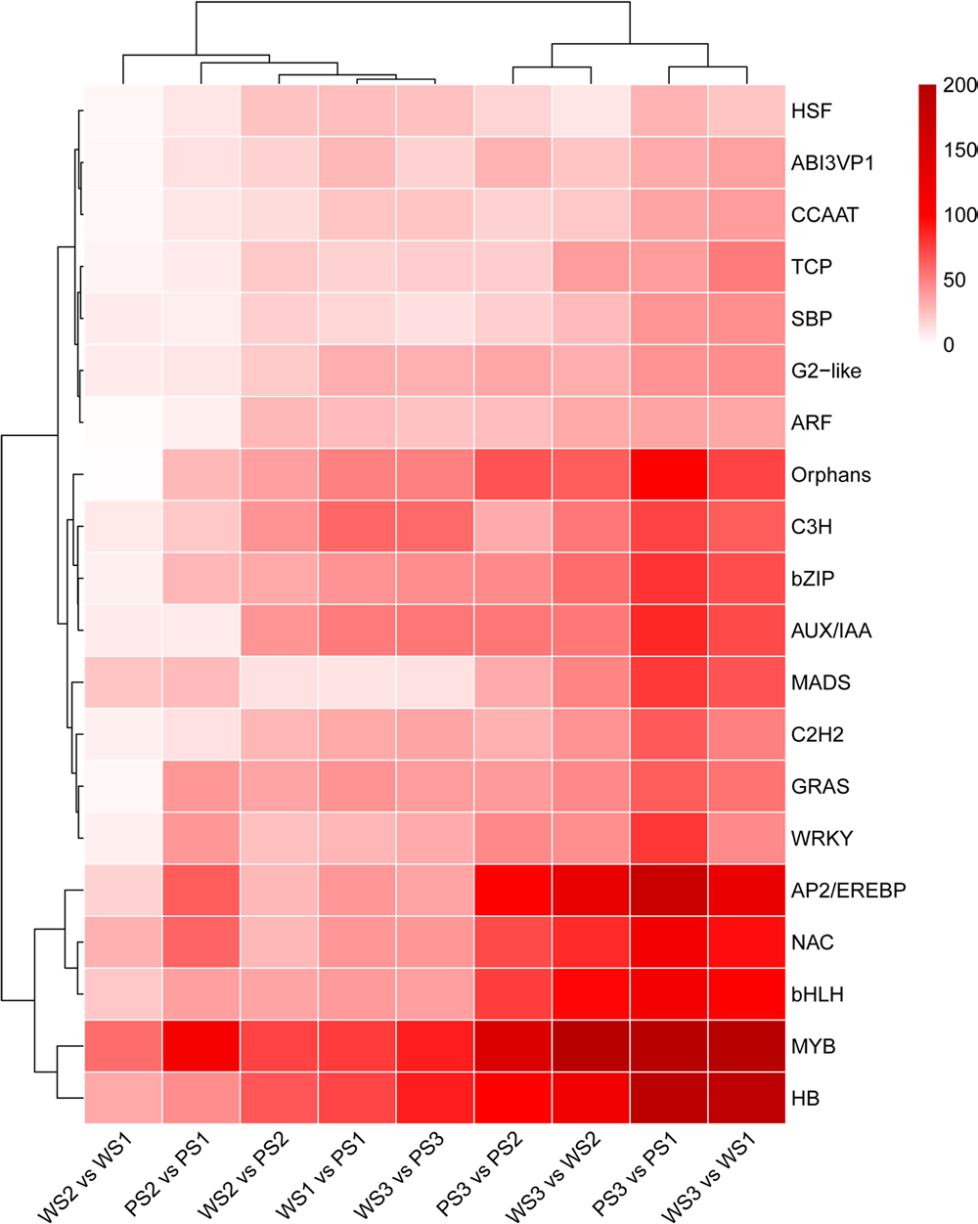
Cluster analysis of the top 20 transcription factor genes differentially expressed at three developmental stages of *L. angustifolia* spike in two cultivars. PS1, PS2, and PS3 indicate the purple lavender flowers at Bud I, Bud II, and flowering stage 50%, respectively. WS1, WS2, and WS3 indicate the white lavender flowers at Bud I, Bud II, and flowering stage 50%, respectively, and 1, 2, and 3 represent three independent biological replicates.

A number of TFs have been reported to play important roles in the synthesis and metabolism of secondary metabolites. In this study, 5732 putative TFs encoding genes were differentially expressed during the process of flowering in lavender, which belonged to 77 major TF families (Supplementary Table S5). The heatmap of the top 20 TFs revealed that most TF members were MYB (1194), followed by HB (910), AP2/EREBP (725), NAC (568), bHLH (566), and Orphans (476) (Fig. 6). An expression heatmap revealed that many TFs had similar expression profiles with genes related to EO pathway (Supplementary Fig. S6), suggesting that these TFs may regulate expression changes of genes involved in the process of terpenoid biosynthesis during inflorescence development.

### Validation of the expression pattern of transcripts via quantitative real-time PCR (qRT-PCR)

**Figure 7:**
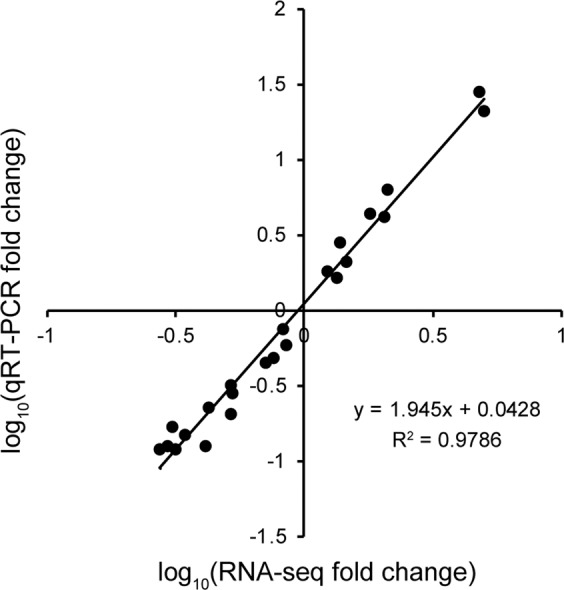
Correlation analyses of fold change values obtained from qRT-PCR and RNA-Seq. RNA-Seq fold change means the ratios of RPKM values of three developmental stages of spikes in two cultivars for selected genes, and qRT-PCR fold change is the relative quantity of the select genes normalised to the expression level of *β-actin*.

To detect the expression levels of putative genes in RNA-Seq data, gene transcription analyses by qRT-PCR were performed for the identified transcripts related to EO synthesis pathways at three different developmental stages between two cultivars. The expression of 17 transcripts was detected, including four genes involved in the MEP pathway, six genes involved in the MVA pathway, and seven genes involved in terpene biosynthesis. As expected, we the expression levels of the 17 genes detected by qRT-PCR showed similar expression patterns as that observed in the transcriptomic data. Experimental results demonstrated that the values of fold change between qRT-PCR and RNA-Seq exhibited a strong positive correlation (R^2^ = 0.9786) according to the linear regression analysis (Fig. 7). These analyses demonstrated that the gene expression values obtained from RNA-Seq were reliable in this study.

## Discussion

After nearly 50 years of development, the planting area of lavender in Xinjiang now amounts to more than 3300 hm^2^, accounting for approximately 95% of the total planting area of lavender in China. In the Yili area in recent years, the two main cultivars are Xinxun 2 (purple flowers) and YXA-5 (white flowers). YXA-5 has a higher oil yield, whereas Xinxun 2 is more popular in production practice because of its high quality EO and ornamental value. In this study, three developmental stages of flower samples were selected for EO extraction according to Lane’s report^9^. GC-MS analysis revealed that the contents of linalool and linalyl acetate increased with similar trends during inflorescence development in each cultivar. The comparison between the two cultivars showed that the contents of linalool and linalyl acetate in Xinxun 2 was higher than that of YXA-5, but the oil rate of YXA-5 was higher than of Xinxun 2 (Fig. 1), suggesting the complexity of EOs biosynthesis during inflorescence development. Therefore, understanding the expression profiles of genes related to EO biosynthesis pathway is interesting during inflorescence development of local cultivars.

RNA-Seq has been applied effectively in the discovery of genes related to terpene biosynthesis in rose-scented geranium (*Pelargonium* sp)^29^ and numerous other plants. However, RNA-Seq studies in EO biosynthesis in lavender species are still limited. Although many genes associated with the EO pathway in this plant have been reported^9,18,19,30^, the molecular mechanisms underlying EO biosynthesis in lavender needs to be further elucidated. In this study, we applied the RNA-Seq approach to explore the transcriptome dynamics in two local lavender cultivars at three developmental stages in spikes and investigated the expression differences in genes related to EO synthesis. In comparison with other published transcriptome including several aromatic plants^29,31,32^, less than 80% SCO revealed that the assembly quality of lavender transcriptome could be further improved. In view of the complexity of lavender genome, our transcriptome data is acceptable. Malli and colleagues described the first *de novo* draft genome of *L. angustifolia* (Maillette) and revealed that lavender genome contains a large proportion of duplicated genes, suggesting past genome polyploidization^15^. We can’t rule out that these two cultivars, Xinxun 2 and YXA-5, are ployploid lines. Or, they are likely two genetic heterogeneities, resulting in possible poor assembly. However, the available transcriptome makes it possible to identify genes associated with EO pathway. Over 73% of the transcripts were annotated in the public database, which constitutes lavender genome resources that facilitates the identification of EO-related genes.

In plants, terpenoids are synthesized mainly through the cytosolic MVA pathway and the plastidial MEP pathway (Fig. 5a). In this study, a total of 27 unigenes associated with the MEP pathway were differentially expressed across different developmental stages of the flower (Table 1). The condensation of pyruvate and G3P is catalysed by DXS, which is transformed into MEP by DXR. We identified seven *LaDXS* transcripts based on BLAST and gene annotation results, which is not much compared with lavender genome (13 copies of *DXS*^15^). Among the seven *DXS* genes, most of them were significantly upregulated at the flowering stage 50% (Fig. 5b; Supplementary Fig. 3), suggesting that the expression levels of *DXS* genes had a similar trend with the biosynthesis of terpenoids during inflorescence development of lavender. Of plant *DXS* family genes, *DXS2* clade was proposed to be involved in isoprenoid biosynthesis^32^. The expression levels of *LaDXS2-1* and *LaDXS2-2* were significantly upregulated from Bud I to flowering stage 50% suggested potential functions in monoterpene biosynthesis, i.e. at least two copies of *DXS* may participate in the same catalytic step. Summarizing the expression profiles of these 27 genes involved in the MEP pathway, we found that the number of upregulated genes was more than the number of downregulated genes, indicating that EO production may increase with the development of flowers in lavender, which was consistent with the measure of EO production (Fig. 1I). Also, these data suggested that monterpenes may produce more through the MEP pathway, and their contents may increase with the maturity of flowers.

The transcriptome examination revealed that 22 transcripts involved in the MVA pathway were differentially expressed across the three developmental stages of the spikes (Table 1). Among the 22 transcripts, seven were annotated as *HMGR*, indicated that multiple copies of *HMGR* homologs were involved in the regulatory step of MVA pathway, while genome contains six copies of *HMGR*^15^. A surprising finding was that all seven *HMGR*, representing a regulatory step of MVA pathway, were downregulated during inflorescence development. Seven *HMGRs* were generally downregulated at flowering stage 50% (Fig. 5b). To our surprise, most of genes involved in the MVA pathway were upregulated during inflorescence development. The concurrently downregulated expression pattern in the MVA pathway suggested that these genes play important roles in the initial stages of lavender flower development; also indicated that compared with the MEP, the MVA pathway is not the main pathway for EO production in lavender spike (Fig. 1).

In rose-scented geranium leaf transcriptome, 13 genes associated with *HMGR* and nine genes related to *DXS* were identified to be highly expressed in leaves^29^. Similarly, five *DXS* were identified to be differentially expressed during flower development in *H. coronarium* flower transcriptome^32^. The putative genes involved in the MEP and MVA pathways in rose-scented geranium and *H. coronarium*, as well as in *L. angustifolia*, exhibited relatively higher expression, which is in agreement with the abundance of EO contents in these plant species. Among these terpene-producing plant species, terpenoid synthesis has a similar pathway, but the difference of gene number and expression levels ultimately affects the quantities and chemical compositions of EOs in each species. In view of this, the identification of the potential genes involved in terpenoid biosynthesis and their expression levels in this study will be helpful to explore the molecular mechanism of EO metabolism in lavender.

Biosynthesis of *Lavandula* terpenes include mono, sesqui, di and triterpenes, which are catalysed by all kinds of terpene synthases^28^. Gene expression analysis through RNA-Seq from several aromatic plants revealed that genes related to terpene biosynthesis displayed a parallel expression pattern with the emission of some monoterpenes, indicating its regulatory role in monoterpene biosynthesis during flower development^2,29,31,32^. Here, two *LaGPPS*, one *GGPPS*, two *rLinS* were upregulated from Bud I to flowering stage 50%, and the expression pattern coincided with the production of EOs in two cultivars (Figs. 1g,h and 5b; Supplementary Fig. 3).

TF plays an important role in the regulation of gene expression related to secondary metabolism. Obviously, not all of the identified TFs are involved in the regulation of EO biosynthesis. Two cultivars, Xinxun 2 and XYA-5, are different in spike color (purple vs white). It is not difficult to imagine that some of these TFs are involved in the anthocyanin biosynthesis. The functional categories of these TFs need further study. The TFs belonging to MYB, bHLH, WRKY, C2H2, C2C2-YABBY, and AP2-EREBP may involve in the regulation of terpenoid biosynthesis. However, the exact roles of many TFs in regulating expression of genes involved in secondary metabolism are unclear. Evidence from *Arabidopsis* has demonstrated that MYC2, a basic helix-loop-helix (bHLH) TF, directly binds to promoters of the sesquiterpene synthase genes TPS21 and TPS11 and activated their expression^33^. However, MYC2 could also directly interact with REPRESSOR OF GA1-3, one of DELLA proteins, and negatively regulate the expression of sesquiterpene synthase genes. In this study, TF analysis revealed that 77 major TF families were differentially expressed from Bud I to flowering stag 50%. Among them, MYB, HB, AP2/EREBP, NAC and bHLH were the most abundant TF members (Fig. 6). These TFs were also identified to be predominantly expressed at different flower developmental stages in *H. coronarium*^32^. In addition, the expression profiles of many TF genes were consistent with those of genes related to EO synthesis, further suggesting their impossible involvement in terpenoid biosynthesis (Fig. 5b; Supplementary Figs. S3 and S6).

In this study, two local cultivars with different EO production were selected for RNA-Seq analysis. The above analysis focused on the expression dynamics of genes within lavender cultivars. In the results, we have described in detail the expression profiles of genes during three development stages from initial flower bud to flowering stage 50% within and across the cultivars, so what was the correspondence between the expression pattern and EO production? It was clear that most genes in the MEP pathway were strongly upregulated at flowering stage 50%, while most genes in the MVA pathway were downregulated in two cultivars (Supplementary Table S4), so we assumed that genes in MEP pathway contribute more the EO production. We hypothesize that cultivars with a higher EO content will present a higher expression of genes associated with the MEP pathway. Specifically, we think that genes that encode proteins such as *DXS*, *HDR* and *LinS* will present the highest expression across the genes of this pathway. As an enzyme that catalyzes the first reaction, DXS is a rate-limiting enzyme and plays crucial role in the MEP pathway^13^. Three *DXSs* were expressed at highest levels at flowering stage 50% in white flower cultivar (YXA-5), while two *DXSs* peaked at flowering stage 50% in purple flower cultivar (Xinxun 2). HDR catalyzes the last reaction of MEP, and five gene encoding HDR were significantly upregulated during inflorescence development in YXA-5, while only two *HDRs* were upregulated in Xinxun 2 (Fig. 5b; Supplementary Fig. 3). It is likely that having more numbers and higher expression levels of the first and last genes allows plants to produce terpenoids more efficiently, which is also partially confirmed by genome assembly analysis^15^. *R*-Linalool contributes most to lavender EO production, but lavender genome contains only two copies of rLinS, and is therefore expected to be strongly expressed in flowers^15^. In YXA-5 with higher EO content, one *rLinS* was very strongly expressed at flowering stage 50%, and its expression levels was significantly higher than that of Xinxun 2 with lower EO content during the same period. Our analysis showed that high abundance of DXS, HDR and rLinS heavily concentrated in YXA-5, leading to a higher content of EOs in YXA-5 than in B. These results confirmed that expression levels of genes related to terpenoid biosynthesis correspond to the content of EO production.

In summary, here we comprehensively analysed the expression profiles of the 77 genes involved in the MEP, MVA, monoterpene and sesquiterpene during inflorescence development in *L*. *angustifolia*. Most of them had similar expression profiles during flower development in two cultivars. The different expression patterns of genes in two cultivars indicated that the process of terpene biosynthesis was complex in lavender species. The expression dynamics of the genes identified in this study might provide a potential explanation for the differential accumulation of EO among varieties. The obtained unigenes and their transcriptional profiles, especially for genes involved in the EO synthesis pathways, would be useful in understanding the molecular basis underlying EO biosynthesis and also facilitate the future engineering of terpene biosynthesis in lavender.

## Conclusion

Using RNA-Seq technology, we *de novo* assembled *L*. *angustifolia* transcriptome data of flower of three development stages from two local cultivars and identified 100,177 DETs in each stage of spike development within and across the cultivars. The largest number of unigenes was involved in “metabolic processes”, including “terpenoid backbone biosynthesis” and “sesquiterpenoid and triterpenoid biosynthesis”. The transcriptome of *L. angustifolia* contains 77 transcripts with annotations related to EO synthesis were differentially expressed across three developmental stages of flowering examined in this study. The upregulated genes were mainly concentrated in the MEP pathway, while most genes in the MVA pathway were downregulated during flower development, and cultivars with a higher EO content presented higher expression of genes related to the MEP pathway, indicating that EOs were chiefly produced through the MEP pathway. Additionally, MYB, HB, AP2/EREBP, NAC, and bHLH constituted the largest number of transcripts encoding TFs in all samples, suggesting their potential functions in regulating EO biosynthesis. The lavender transcript database offers a useful resource for studying terpene biosynthesis in plant.

## Methods

### Plant materials, cultivation and sample collection

Two *L. angustifolia* cultivars with different flower colours, Xinxun 2 and YXA-5, were used in this study. These two varieties have been bred for many years, and have become the main local varieties. The colour of verticillaster inflorescence is purple for Xinxun 2 and is white for YXA-5 (Fig. 1, Supplementary Fig. 3). Lavender cuttings were planted in the Resources Nursey experimental field at the Institute of Agricultural Sciences, Fourth Division of Yili of Xinjiang Production and Construction Corps in Xinjiang (620 m above sea level: 43° 92′N, 81° 32′E) during autumn season of 2015.

The flower tissues at three developmental stages were harvested from two cultivars according to the length of the spike (cm) and number of open flowers per plant during spring season of 2018 (Fig. 1). Bud I included flower buds ≤0.5 cm in length; Bud II included flower buds ≤0.5–1 cm in length. At the flowering stage 50%, 50% of individual flowers per flower spike were opened. Samples of each period were collected from six independent individuals for RNA-Seq analysis and EO extraction, respectively. Samples collected from Xinxun 2 were named PS1, PS2 and PS3; samples collected from YXA-5 were named WS1, WS2 and WS3. The harvested samples were frozen in liquid nitrogen immediately and store −80 °C until use. With three biological replicates, altogether 18 samples were used for total RNA isolation in this study.

### Extraction of EO and GC-MS analysis

The extraction of lavender EO from *L. angustifolia* flowers was achieved by steam distillation as previously reported^1,30^. Approximately 0.5 g of lavender flowers were vigorously mixed with 2 mL n-hexane and then placed in a 5 L distilling vessel (bottom flask). Water was added until the plant materials were submerged. From when the first drops of EO appear, the distillation ended after boiling for 50 minutes. The EOs were collected after 10 minutes in an oil-water separator. The water was precipitated with anhydrous sodium sulfate, and pure EOs were collected after centrifugation. A mixture of 500 mL pure EOs and 500 mL n-hexane was detected by GC-MS analysis. Each extraction was performed at least three times. The content of each terpene was calculated as percentage of the total EOs. Oil rate was calculated by dividing the oil weight by the fresh weight.

### Illumina sequencing, quality control, transcriptome assembly and gene annotations

For RNA extraction and quantification, library construction, and RNA-Seq for each sample were carried out as described by Yang *et al*.^34^. Sequencing libraries were constructed using NEBNext Ultra II RNA Library Prep Kit for Illumina (NEB, USA) following manufacturer’s protocols. The cycles of PCR for cDNA amplification were 10 in this study. The insert sizes of the library fragments were 150~200 bp. All 18 libraries (six samples in three biological replicates) were sequenced on an Illumina HiSeq. 4000 platform at the Novogene Bioinformatics Institute (Beijing, China), and raw Illumina paired-end reads were generated, which were first processed using in-house Perl Scripts^34^ with the parameter ‘-q 33 -t 4 -L 20 -N 0.001’. On removing adaptors, poly-N and low-quality reads from raw data, clean reads were obtained and their Q20, Q30, GC content and sequence duplication level were calculated. *De novo* assembly of the transcriptome was accomplished using default parameters in the Trinity programme version v2.4.0^25^. Completeness of the assembled genome was assessed by performing core gene annotation using the BUSCO v3.0.2^27^ methods.

Gene expression levels were identified by RSEM^25^ (https://github.com/deweylab/RSEM) for each sample and genes with FPKM > 0.3 from samples were used for further analysis^35,36^. Sequences were annotated by conducting BLAST searches against public databases, including the NCBI (https://www.ncbi.nlm.nih.gov/) non-redundant nucleotide (NT), NR, Swiss-Prot protein (http://www.expasy.org/sprot/, *E*-value ≤ 1e-5), cluster of orthologous groups (http://www.ncbi.nlm.nih.gov/COG/, *E*-value ≤ 1e-3), Pfam (http://pfam.xfam.org/, *E*-value ≤ 0.01), and KEGG (http://www.genome.jp/kegg, *E*-value ≤ 1e-10). According to the NR annotations, the Blast2GO program^37^ (http://www.blast2go.com) was used to annotate GO (http://www.geneontology.org) terms (*E*-value ≤ 1e-6).

### Identification of DEGs and putative TF-related genes from lavender transcriptome

In this study, we used the DESeq2R package (v1.10.1)^35^ to analyse the differentially expressed transcripts between two phases within and across two lavender cultivars at three different developmental stages. The analysis methods of DEGs and TFs were the same as that of Yang *et al*.^34^, so we will not elaborate on this here. A brief description is as follows: using the *p.adjust* function to adjust the resulting *P*-values to control the false discovery rate; genes with an adjusted *P*-value less than 0.05 were considered to be differentially expressed; taking an absolute value of log_2_(Group1/Group2) ≥ 1 as the threshold for judging significant DEGs between different developmental stages; using the GOseq R packages^34^ to conduct GO enrichment analysis; and using the KOBAS^38^ software (v2.0.12) to analyse the KEGG enrichment pathway of DEGs.

### qRT-PCR validation

Validation of the results of RNA-seq was carried out by qRT-PCR. qRT-PCR analyses were performed as described by Yang *et al*.^34^. The gene-specific primers used in this study are shown in Supplementary Table S6. *β-actin* was used as an internal ref. ^2^. The 2^−∆∆CT^ method was used to calculate the relative gene expression levels^39^. For each gene, three technical and biological replicates were used in the qRT-PCR experiment.

## Supplementary information

Supplementary information.

Supplementary information2.

Supplementary information3.

## Data Availability

All RNA-Seq raw data used in this study were deposited in the Sequence Read Archive (SRA) at NCBI under accession number SRP158322.
