# Supplementary material for: Transcriptome profiling of spike provides expression features of genes related to terpene biosynthesis in lavender
Source: Sci Rep. 2020 Apr 24;10:6933. doi: 10.1038/s41598-020-63950-4 (PMC7181790; doi:10.1038/s41598-020-63950-4)
Supplement: Supplementary file 1 — Supplementary information. [file 41598_2020_63950_MOESM1_ESM.pdf]

Transcriptome profiling of spike provides expression features of genes related to terpene biosynthesis in lavender

Danli Guo[1,2,+], Kaicheng Kang[2,+], Pu Wang[1], Min Li[1,*], Xianzhong Huang[2,3,*]

[1]Institute of Agricultural Sciences, Fourth Division of Xinjiang Production and Construction Corps, Cocodala, 835213, People's Republic of China

[2]Special Plant Genomics Laboratory, College of Life Sciences, Shihezi University, Shihezi, 832003, People's Republic of China

[3]College of Agriculture, Anhui Science and Technology University, Fengyang, 233100, People's Republic of China

[*]Correspondence: Xianzhong Huang (xianzhongh106@163.com); Min Li (1341997078@qq.com)

[+]these authors contributed equally: Danli Guo, Kaicheng Kang

Supplementary Information

Supplementary Fig. S1. Comparison of gene expression levels under different spike developmental stages in two cultivars.

Supplementary Fig. S2. Gene ontology (GO) enrichment analysis of differentially expressed genes (DEGs) during *L. angustifolia* flower development.

Supplementary Fig. S3. Differential expression of the unigenes related to the essential oil (EO) biosynthesis pathway in Xinxun 2.

Supplementary Fig. S4. A neighbour-joining tree based on amino acid sequences of LaHMGR and selected plant HMGR sequences.

Supplementary Fig. S5. Amino acid sequences alignment of LaPPS and selected plant PPS sequences.

Supplementary Fig. S6. Differential expression of the unigenes encoding putative transcription factors.

Supplementary Fig. S7. Field grown *Lavandula angustifolia*.

Supplementary Table S1. Overview of sequence data quality obtained from Illumina sequencing.

Supplementary Table S2. The transcriptome quality of the *L. angustifolia* assembly were aligned to conserved genes using BUSCO method.

Supplementary Table S3. KEGG pathway analysis of DEGs among different comparisons during the different developmental stages of spike in two *L. angustifolia* cultivars

Supplementary Table S4. Information for differentially expressed genes related to terpene biosynthesis in *L. angustifolia*.

Supplementary Table S5. Information for differentially expressed transcription factors during the developmental stages of spikes in *L. angustifolia*.

Supplementary Table S6. Primers used in qRT-PCR validation in this study.

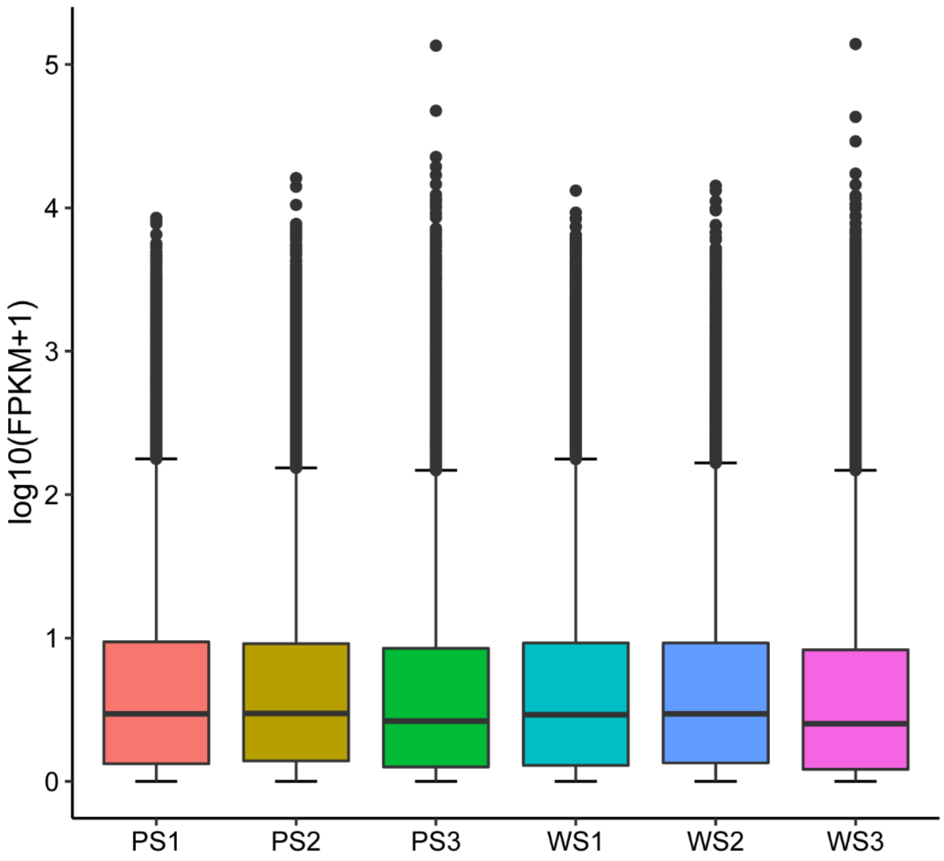

Supplementary Fig. S1. Comparison of gene expression levels at different spike developmental stages in two cultivars. Boxplot showing the distribution of FPKM values at three stages in two cultivars. The x-axis in the boxplot is the sample name. The y-axis is the log_{10}(FPKM+1). PS1, PS2 and PS3 indicate the purple lavender flowers at Bud I, Bud II, and flowering stage 50%, respectively. WS1, WS2 and WS3 indicate the white lavender flowers at Bud I, Bud II, and flowering stage 50%, respectively.

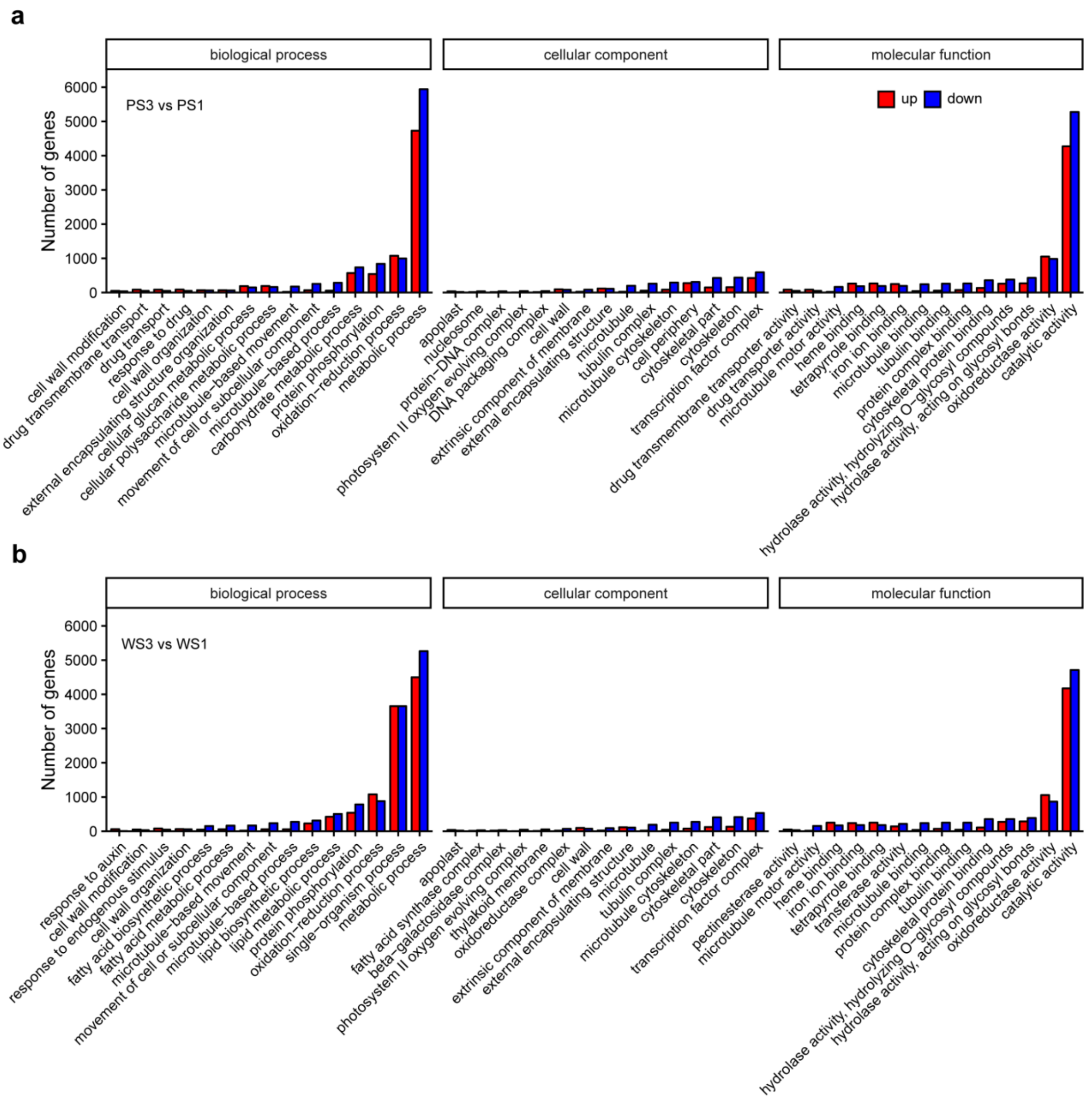

Supplementary Fig. S2. Gene ontology (GO) enrichment analysis of differentially expressed genes (DEGs) during *L. angustifolia* flower development. a Comparison between PS1 and PS3. PS1 and PS3 indicate purple flower development in Xinxun 2 at Bud I and flowering stage 50%, respectively. **b** Comparison between WS1 and WS3. WS1 and WS3 indicate white flower developments of YXA-5 at Bud I and flowering stage 50%, respectively. The x-axis shows the GO classification of the *L. angustifolia* transcriptome. The y-axis shows the number of genes in a specific category.

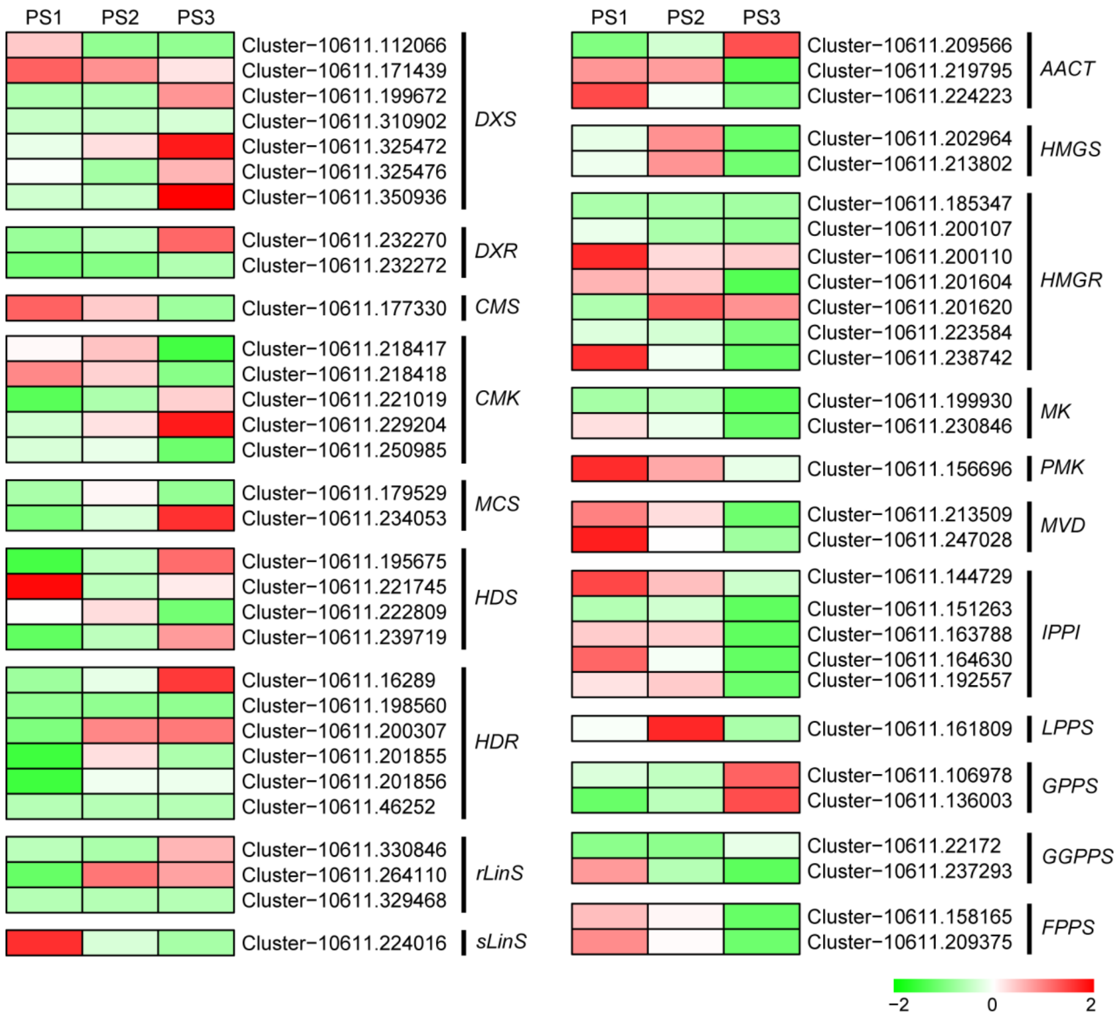

Supplementary Fig. S3. Differential expression of the unigenes related to the essential oil (EO) biosynthesis pathway in Xinxun 2. Heatmap depicting the expression profile of EO biosynthesis-related genes involved in the different reaction steps. The details of the transcripts are given in Supplementary Table S4.

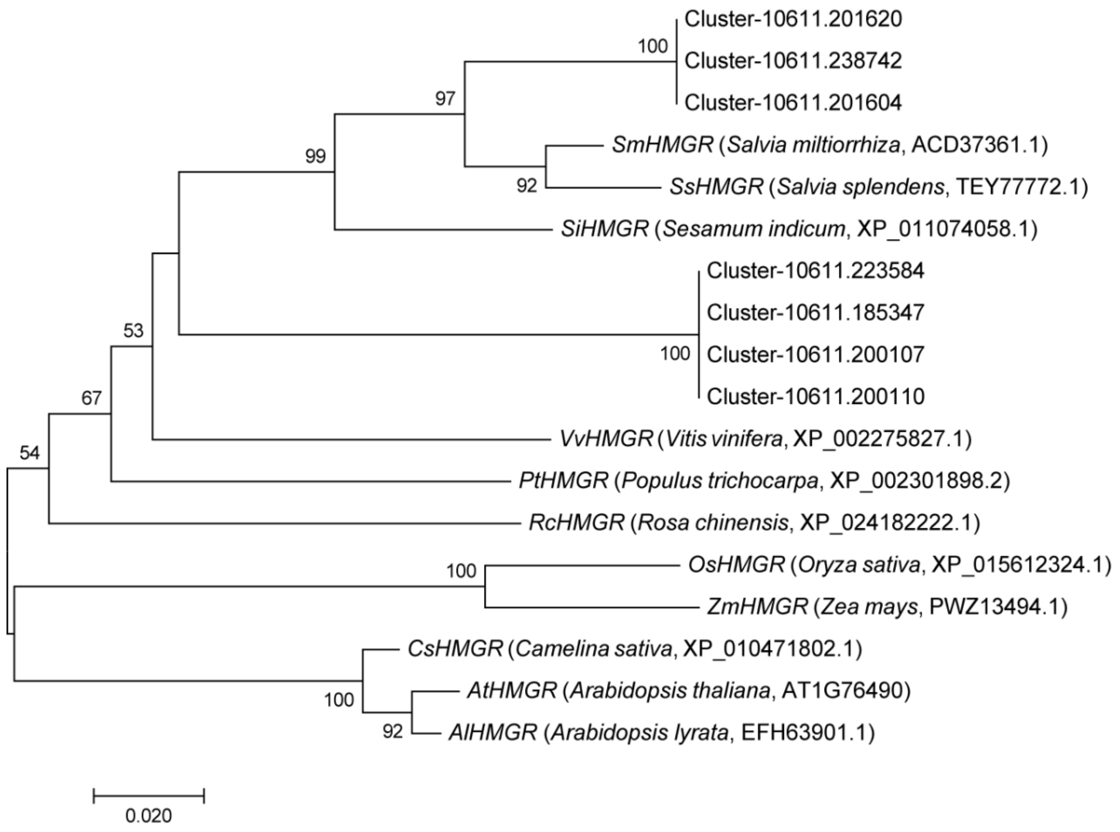

Supplementary Fig. S4. A neighbour-joining tree based on amino acid sequences of LaHMGR and selected plant HMGR sequences.

The tree was generated using Phylogeny Analysis MEGA7.0 program (Kumar et al., 2016).

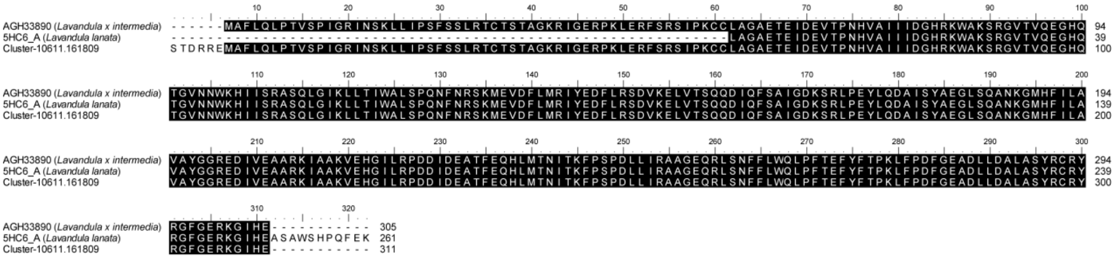

Supplementary Fig. S5. Amino acid sequences alignment of LaPPS and selected plant PPS sequences.

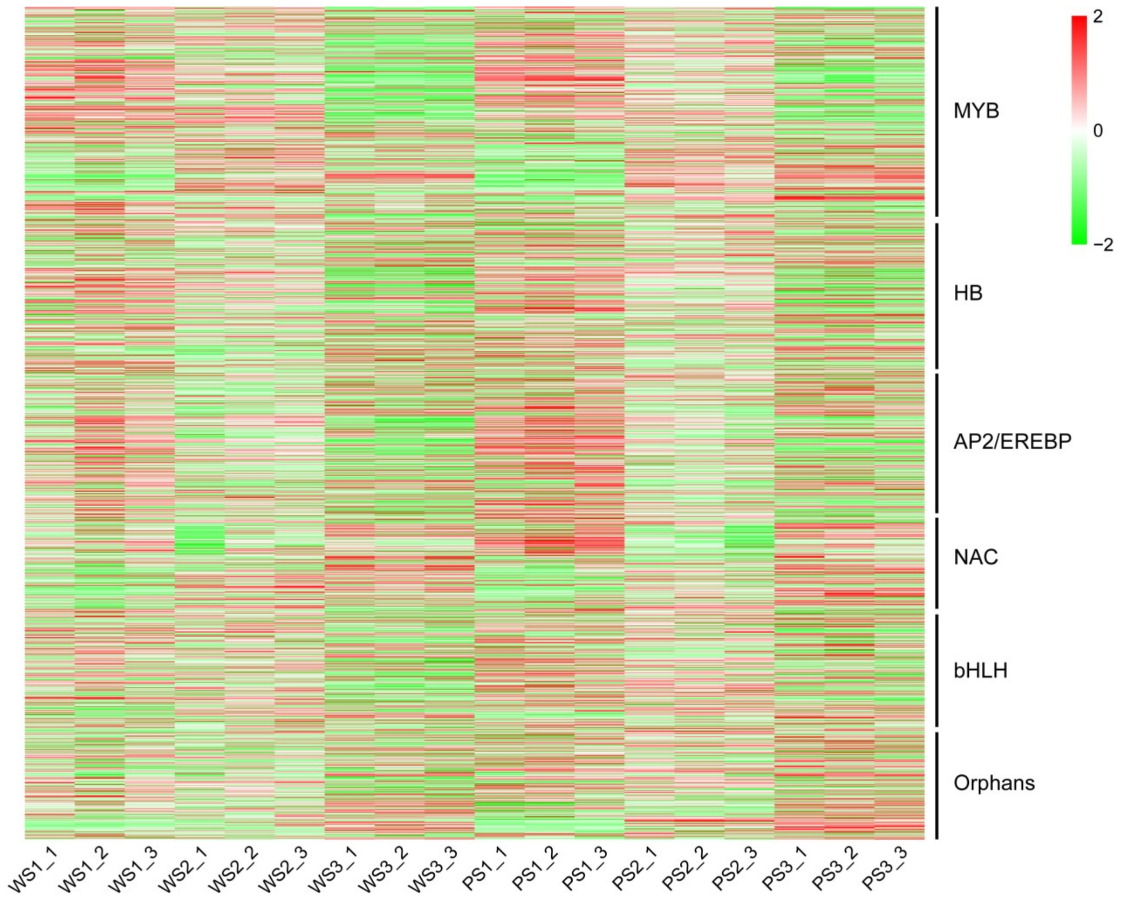

Supplementary Fig. S6. Differential expression of the unigenes encoding putative Transcription factors.

PS1, PS2, and PS3 indicate the purple lavender flowers at Bud I, Bud II, and flowering stage 50%, respectively. WS1, WS2, and WS3 indicate the white lavender flowers at Bud I, Bud II, and flowering stage 50%, respectively, and 1, 2, and 3 represent three independent biological replicates.

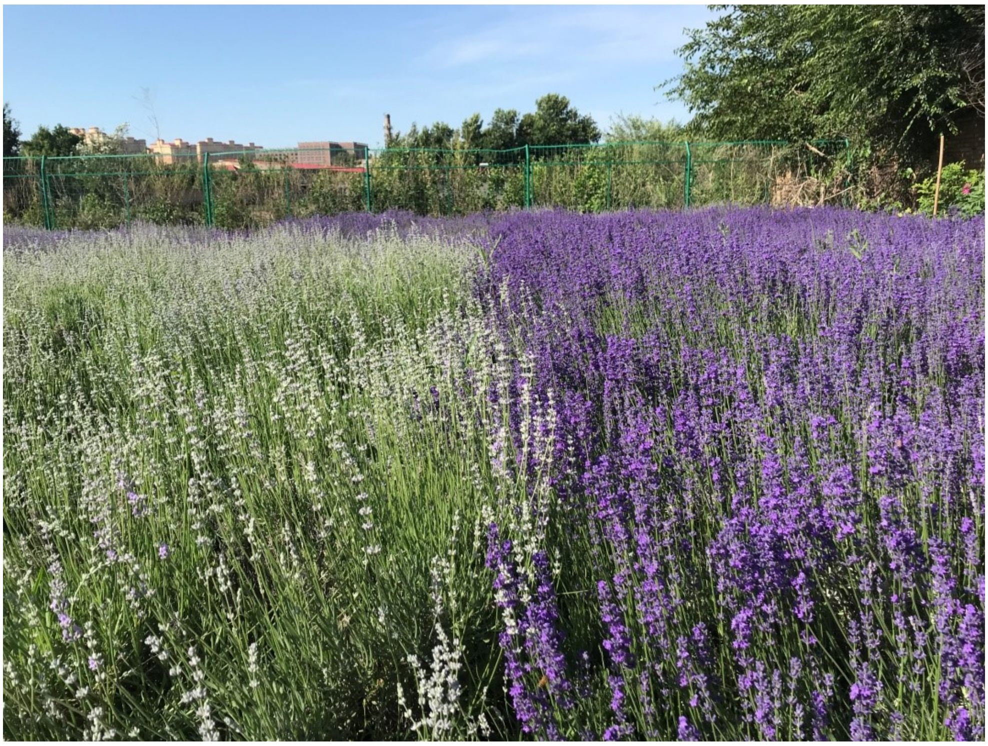

Supplementary Fig. S7. Field grown *Lavandula angustifolia*. The white flowers on the left are YXA-5, and the purple flowers on the right are Xinxun 2.

References

Kumar S, Stecher G, Tamura K (2016) MEGA7: molecular evolutionary genetics analysis version 7.0 for bigger datasets. Mol Biol Evol 33:msw054. https://doi.org/10.1093/molbev/msw054.

Supplementary Table S1. Overview of sequence data quality obtained from Illumina sequencing.

Sample	Raw Reads	Clean Reads	Clean Bases	Error (%)	Q20 (%)	Q30 (%)	GC Content (%)
PS1_1	64268510	62859224	9.43G	0.03	96.84	95.12	47.94
PS1_2	60264654	59762062	8.96G	0.03	96.5	94.76	47.52
PS1_3	57497558	56525652	8.48G	0.03	96.23	94.38	47.7
PS2_1	65906608	65029806	9.75G	0.03	96.63	94.81	48.07
PS2_2	45092874	43878598	6.58G	0.03	96.48	94.71	47.42
PS2_3	50525652	49718840	7.46G	0.03	97.12	95.45	47.5
PS3_1	64987312	64371512	9.66G	0.03	96.74	94.97	47.67
PS3_2	51331134	50740120	7.61G	0.03	96.44	94.67	47.42
PS3_3	62626000	62021286	9.3G	0.03	96.78	95.03	47.68
WS1_1	68059712	67349530	10.1G	0.03	96.82	95.1	48.02
WS1_2	79801882	78899312	11.83G	0.03	96.18	94.17	47.56
WS1_3	69297328	68326122	10.25G	0.03	96.49	94.6	47.55
WS2_1	44216630	43570600	6.54G	0.03	96.93	95.25	47.78
WS2_2	42244632	41606902	6.24G	0.03	96.24	94.4	47.5
WS2_3	43799742	43375404	6.51G	0.03	96.37	94.58	47.53
WS3_1	65602270	64685530	9.7G	0.02	97.28	95.7	47.54
WS3_2	47538098	46807660	7.02G	0.03	96.57	94.84	47.13
WS3_3	47108990	46216630	6.93G	0.03	96.7	95.03	47.45
**Total**	**1030169586**	**1015744790**					

PS1, PS2, and PS3 indicate the purple Lavender flowers at 'Bud I', 'Bud II', and flowering stage 50%, respectively.

WS1, WS2, and WS3 indicate the white Lavender flowers at 'Bud I', 'Bud II', and flowering stage 50%, respectively.

Supplementary Table S2. The transcriptome quality of the *L. angustifolia* assembly were aligned to conserved genes using BUSCO method.

Type	Number	Percent (%)
Complete BUSCOs	1064	73.9
Complete and single-copy BUSCOs	202	14.0
Complete and duplicated BUSCOs	862	59.9
Fragmented BUSCOs	215	14.9
Missing BUSCOs	161	11.2
Total BUSCO groups searched	1440	100

Supplementary Table S3. KEGG pathway analysis of DEGs among different comparison during the different developmental stages of spike in two *L. angustifolia* cultivars.

KEGG Pathway	PS2/PS1	PS3/PS2	PS3/PS1	WS2/WS1	WS3/WS2	WS3/WS1
1. Starch and sucrose metabolism	137	242	354	58	293	306
2. Plant hormone signal transduction	106	359	499	33	403	441
3. Cysteine and methionine metabolism	99	125	152	42	154	130
4. Phenylpropanoid biosynthesis	72	139	191	86	190	200
5. Pentose and glucuronate interconversions	58	152	201	23	160	197
6. Flavonoid biosynthesis	57	33	71	43	69	89
7. Carotenoid biosynthesis	32	26	71	15	41	42
8. Tyrosine metabolism	32	51	88	19	69	92
9. Photosynthesis-antenna proteins	29	105	52	0	102	98
10. Cutin, suberine and wax biosynthesis	26	88	102	26	96	103
11. Isoquinoline alkaloid biosynthesis	26	42	74	13	52	72
12. Terpenoid backbone biosynthesis	26	87	133	6	130	117
13. Ubiquinone and other terpenoid-quinone biosynthesis	22	34	61	21	52	54
14. Glyoxylate and dicarboxylate metabolism	20	101	100	44	141	78
15. Sesquiterpenoid and triterpenoid biosynthesis	19	40	43	10	52	30
16. Stilbenoid, diarylheptanoid and gingerol biosynthesis	18	43	42	23	59	65

17. Diterpenoid biosynthesis	17	38	46	1	48	50
18. Flavone and flavonol biosynthesis	16	4	17	5	14	20
19. Ascorbate and aldarate metabolism	16	78	77	7	97	84
20. Fatty acid degradation	15	74	101	7	90	99

PS1, PS2, and PS3 indicate the purple Lavender flowers at 'Bud I', 'Bud II', and flowering stage 50%, respectively.

WS1, WS2, and WS3 indicate the white Lavender flowers at 'Bud I', 'Bud II', and flowering stage 50%, respectively.

Supplementary Table S6. Primers used in qRT-PCR validation in this study

Gene	Gene id	Primer name	Primer sequence
La-β-actin	Cluster-10611.208954	La-β-actin-F	TGTGGATTGCCAAGGCAGAGT
		La-β-actin-R	AATGAGCAGGCAGCAACAGCA
LaDXS1	Cluster-10611.354968	LaDXS1-F	CATTCCTCCCACCTTCACCT
		LaDXS1-R	TCTCCTTCCTGATCGCCATC
LaMCS	Cluster-10611.215052	LaMCS-F	GCATCTAAGTTCCCAAGCTCG
		LaMCS-R	CCGATGGTGATGTGTTACTGC
LaHDR1	Cluster-10611.201855	LaHDR1-F	TGCCGTTCTTGAGTTGCATC
		LaHDR1-R	GGGACCTTGAGAGAGTTGGT
LaAACT3	Cluster-10611.258286	LaAACT3-F	AATGTTGCTAAGTTCGAGACCAG
		LaAACT3-R	CATCCTCTTAAGAAGTCTGTGCC
LaHMGS3	Cluster-10611.202964	LaHMGS3-F	GAACATCCATTCAGCCTGTCC
		LaHMGS3-R	GGTGAGATAGTACGTCCCTGG
LaHMGR1	Cluster-10611.223584	LaHMGR1-F	CTTGTACCTAACCAACACCGTC
		LaHMGR1-R	AAGATAGATGAAGGAGGCGAGG
LaMK2	Cluster-10611.230846	LaMK2-F	GCTTCCATCGAGACTGTGATAAG
		LaMK2-R	GAAGCTAATCTCCATGCCTCTTC
LaMVD1	Cluster-10611.246401	LaMVD1-F	GTCACGCATTCCAGATGTACTAC
		LaMVD1-R	TGGATTTGTCAAGTGGATCATGG
LaMVD2	Cluster-10611.259971	LaMVD2-F	CCCAGCATCAAAGGTATAAGCC
		LaMVD2-R	AGAGGAAGTAGTACCAAAGCGT
LaIPI2	Cluster-10611.203442	LaIPI2-F	GAATCCGCCTTCTTCAGCAG
		LaIPI2-R	CTACAAAGCACCATCCGACG
LaLPPS1	Cluster-10611.376333	LaLPPS1-F	TGTGGACAGTGAAGAAGCTCT
		LaLPPS1-R	GTTGGCTGCTGTGAGTTTCC
LaGGPPS1	Cluster-10611.174422	LaGGPPS1-F	TCTATCATCCCTCTCACGGTTC
		LaGGPPS1-R	CAACGGTCAAGATTCGAGTTCA
LaGGPPS4	Cluster-10611.198309	LaGGPPS4-F	GTCGCTAGTATCCTCTCCGG
		LaGGPPS4-R	GTCTCTCATCCACGACGACT

LaFPPS1	Cluster-10611.209375	LaFPPS1-F	CCCTCCAGGCACATTGTAGT
		LaFPPS1-R	ATGGCGAATATGAACGGAGC
LaFPPS2	Cluster-10611.158165	LaFPPS2-F	CGTCGTGTGTGTGAGTTATCC
		LaFPPS2-R	CTGCTTTCGAGTGGACTGATG
LaBPPS	Cluster-10611.201987	LaBPPS-F	ACGTCAATGAACCATCTTGCAT
		LaBPPS-R	GGGCGACTTTAGATCAAGCATT
LaS-LinS	Cluster-10611.217256	LaS-LinS-F	CTCAAGATATGGAACGTCGGC
		LaS-LinS-R	TACTTCCTCAGCCAGCTCAAT
